# Upregulated Expression of Macrophage Migration Inhibitory Factor, Its Analogue D-Dopachrome Tautomerase, and the CD44 Receptor in Peripheral CD4 T Cells from Clinically Isolated Syndrome Patients with Rapid Conversion to Clinical Defined Multiple Sclerosis

**DOI:** 10.3390/medicina55100667

**Published:** 2019-10-01

**Authors:** Eugenio Cavalli, Emanuela Mazzon, Maria Sofia Basile, Katia Mangano, Roberto Di Marco, Placido Bramanti, Ferdinando Nicoletti, Paolo Fagone, Maria Cristina Petralia

**Affiliations:** 1 Department of Biomedical and Biotechnological Sciences, University of Catania, 95123 Catania, Italy; eugeniocavalli9@hotmail.it (E.C.); sofiabasile@hotmail.it (M.S.B.); kmangano@unict.it (K.M.); paolofagone@yahoo.it (P.F.); 2 IRCCS Centro Neurolesi Bonino Pulejo, C.da Casazza, 98124 Messina, Italy; emanuela.mazzon@irccsme.it (E.M.); placido.bramanti@irccsmet.it (P.B.); m.cristinapetralia@gmail.com (M.C.P.); 3 Department of Medicine and Health Sciences “Vincenzo Tiberio”, University of Molise, 86100 Campobasso, Italy; roberto.dimarco@unimol.it

**Keywords:** macrophage migration inhibitory factor, d-dopachrome tautomerase, multiple sclerosis, CD4^+^ T cells

## Abstract

*Background and objectives*: Macrophage Migration Inhibitory Factor (MIF) and D-Dopachrome Tautomerase (DDT) are two pleiotropic and primarily, but not exclusively, proinflammatory cytokines belonging to the MIF family of cytokines that have recently been shown to be implicated in the pathogenesis of progressive forms of human progressive Multiple Sclerosis (MS) and the experimental model counterpart in rodents. *Materials and Methods:* We have presently evaluated a transcriptomic analysis of the expression of MIF, DDT, their receptors CD74 and CD44, and MIF co-receptors CXCR2, CXCR4, and CXCR7 in peripheral blood of patients with Clinically Isolated Syndrome (CIS), with rapid progression to clinical defined MS. *Results:* Our analysis reveals that MIF, DDT, and CD44 are overexpressed in CD4^+^ T cells from patients with CIS, as compared to healthy controls. Accordingly, a significant overlap was observed between the genes overexpressed in CD4^+^ T cells from patients with CIS and the genes belonging to the MIF regulatory network. This upregulated expression appeared to be unique for CD4^+^ T cells, as other immune cells including CD8^+^ T cells, B cells, and monocytes from these patients exhibited expression levels of these molecules that were superimposable to those observed in healthy controls. *Conclusions:* Overall, our data suggest that the overexpression MIF cytokine family signature may occur in CD4^+^ T cells from patients with CIS, and that this phenomenon may be implicated in the pathogenesis of the disease, offering the possibility to represent both a diagnostic marker and a therapeutic target.

## 1. Introduction

Macrophage Migration Inhibitory Factor (MIF) and D-Dopachrome Tautomerase (DDT) are two cytokines belonging to the MIF family that possess pleiotropic properties and are implicated in the pathogenesis of a wide arrays of pathologies including autoimmune diseases, cancer, and neurodegenerative disorders [1,2,3]. Both MIF and DDT bind to the same receptor CD74 and for both cytokines the subsequent interaction of CD74 with CD44 is necessary for signal transduction [4,5].

The signal transduction pathway of MIF has been better characterized than that of DDT. It is known that cell signaling through the CD74 and CD44 receptors leads to activation of several signaling pathways including phosphatidylinositol-4, 5-bisphosphate3-kinase (PI3K), protein kinase B (AKT) Extracellular-signal Regulated Kinase (ERK1/2), and 5′ adenosine monophosphate-activated protein kinase (AMPK). The signaling of DDT via the CD74/CD44 complex has also been shown to activate the ERK1/2 signaling pathway [6,7]. MIF has also been shown to bind most often in a CD74-dependent manner with the other co-receptors CXCR2, CXCR4, and CXCR7 that do not seem to be bound from DDT [6,7]. MIF and DDT can also profoundly modulate cell function, proliferation, and growth by directly interfering with intracellular protein such as the c-Jun activation domain-binding protein 1/COP9 signalosome subunit 5 (JAB1/CSN5) that is inhibited by MIF and DDT. MIF and DDT-mediated inhibition of JAB1/CSN5 may explain the cell cycle arrest induced by MIF genetic ablation of MIF or specific pharmacological antagonism in some experimental settings [6,7].

Much attention has recently been focused on the possible pathogenetic role played from an upregulated production and excessive signaling of the MIF cytokine family and their receptors in the demyelinating immunoinflammatory disease multiple sclerosis (MS) and its rodent counterpart experimental autoimmune encephalomyelitis (EAE) [3,8]. In particular, Benedek and coworkers [8] demonstrated that male patients with progressive MS had augmented blood levels of MIF and DDT levels as compared to both females with progressive MS and MS patients with relapsing-remitting forms of the disease. The increased levels of MIF and DDT levels in males significantly correlated with the presence of two high-expression promoter polymorphisms located in the MIF gene, a -794CATT5-8 microsatellite repeat and a -173 G/C SNP [8]. Knocking out of either MIF or DDT rendered the mice less susceptible to EAE development [8]. In a transcriptomic analysis we observed an upregulation of the receptors involved in MIF signaling both in the animal model and in MS patients along with a significant increase in MIF receptors in the CNS lesions associated to MS. In agreement with the data of Benedek and coworkers [8], we demonstrate that negating the endogenous action of MIF with its specific small molecule inhibitor ISO1 ameliorated the course of the disease as compared to vehicle-treated animals [9].

The aim of our present study was to demonstrate whether an increased expression of MIF, DDT, and their receptors occurred in patients suffering from clinically isolated syndrome (CIS), which is an immunoinflammatory demyelinating disease which frequently affects the optic nerve, often preceding MS development [10,11]. For this purpose, we carried out a transcriptomic analysis of MIF, DDT, and their receptors and co-receptors in CIS patients with subsequent rapid development of Clinically Definite Multiple Sclerosis (CDMS).

## 2. Materials and Methods

### 2.1. Generation of a MIF “Regulatory Molecular Network”

For the generation of the MIF “Regulatory Molecular Network”, which captures the current knowledge on the functional modularity and interconnectivity of genes in a cell, the GeneMania database (http://www.genemania.org/) was used [12]. The parameters used were as follow: Automatically selected weighting method; max resultant genes: 20; max resultant attributes: 10. The following categories for gene interconnection were used: Co-expression, Physical and Genetic interactions, Pathways, Co-localization, Shared Protein Domain, and Predicted Interactions.

### 2.2. Microarray Selection

Microarray gene expression data were obtained from the National Center for Biotechnology Information Gene Expression Omnibus (GEO; https://www.ncbi.nlm.nih.gov/geo/). The GSE62584 was selected to assess the expression levels of MIF, and related genes, in (Clinical Isolated Syndrome, CIS) patients with CIS. In particular, the GSE62584 included whole-genome transcriptomic data from Peripheral Blood Mononuclear cells (PBMCs) subpopulations (i.e., CD19^+^ B cells, CD14^+^ macrophages, CD4^+^ and CD8^+^ T cells), from 6 patients with acute onset of the first demyelinating event of ON referable to CIS (4 females, 2 males) and 9 age- and sex-matched healthy subjects [13]. Patients included were between 18–40 years of age; had a diagnosis of CIS based on unilateral visual loss 20/40 that was associated with dyschromatopsia, an afferent pupillary defect, and a central/paracentral visual field defect; had no prior neurological symptomatology; had an MRI indicative of demyelination; had development of definite MS within one year of clinical follow-up. The time from the onset of visual symptoms to blood sampling was ≤96 h [13]. The Affymetrix Human Genome U133A 2.0 Array was used, and raw data was preprocessed by GC Robust Multiarray Analysis (RMA).

### 2.3. Ethical Approval

This study is a third-party re-analysis of the publicly available microarray dataset GSE62584, already published by Feldman and collaborators [13], and approved by the Sheba Medical Center Internal Review Board (IRB) committee.

### 2.4. Statistical Analysis

Unpaired two-tailed Student *t* test was used to determine significance in gene expression differences among healthy controls and CIS patients. Differentially Expressed Genes (DEGs) were selected using the following parameters: *p* value 0.05 and fold change 1.5. Chi-square Test with Yates’ correction was used to assess enrichment significance. *p* values 0.05 were considered to be statistically significant.

## 3. Results

### 3.1. Expression of MIF and Related Genes in CIS Patients

**Figure 1:**
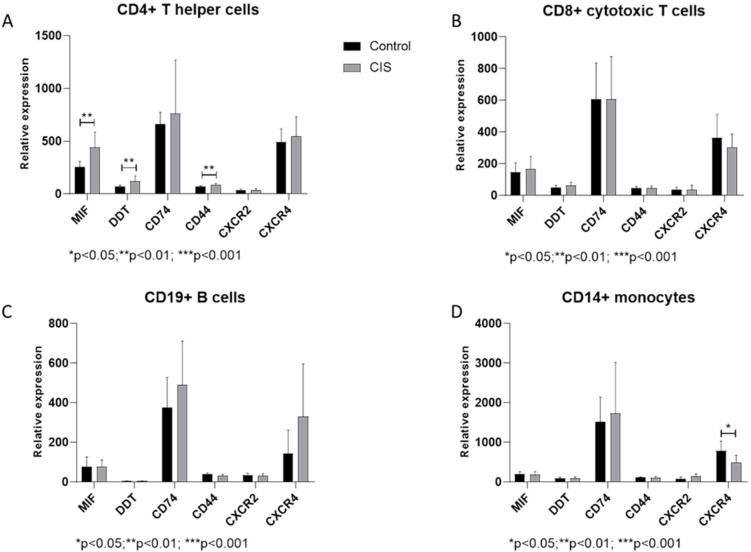
Relative expression levels of Macrophage Migration Inhibitory Factor (MIF), D-Dopachrome Tautomerase (DDT)and related receptors, in CD4^+^ T helper cells (**A**), CD8^+^ cytotoxic T cells (**B**), CD19^+^ B cells (**C**) and CD14^+^ monocytes (**D**), isolated from Peripheral Blood Mononuclear cells (PBMCs) of healthy donors and Clinically Isolated Syndrome (CIS)patients, as determined in the GSE62584 dataset.

As shown in Figure 1A, MIF levels were significantly increased in CD4^+^ T cells from CIS patients, as compared to healthy donors. Along the same lines, DDT results showed significant augmentation (Figure 1A). A moderate but significant increase was also observed for CD44 in CD4^+^ T cells from CIS patients, as compared to healthy donors (Figure 1A). No modulation was instead observed for the other receptors and co-receptors.

No modulation in MIF and DDT levels was observed in the other peripheral immune cell subpopulations, i.e., CD8^+^ T cells, CD19^+^ B cells, and CD14^+^ monocytes (Figure 1B–D). The receptors CD74 and CD44, as well as the co-receptors CXCR-2 and -4, were not significantly different between healthy subjects and CIS patients in the cell populations investigated, with the exception of CXCR4 that resulted significantly reduced in monocytes from CIS patients as compared to controls (Figure 1B–D).

### 3.2. Enrichment of the MIF Regulatory Network in CIS

**Figure 2:**
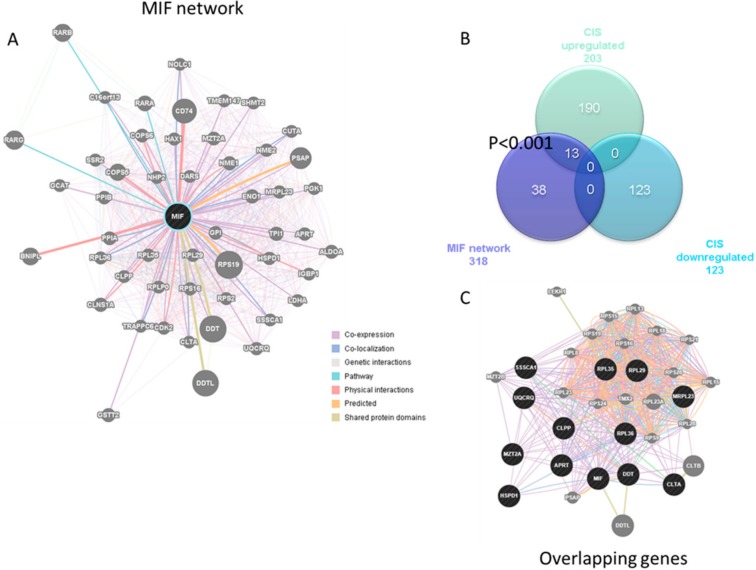
The MIF regulatory network was constructed using the GeneMania web-based utility (**A**), and Venn analysis was used for the determination of its enrichment among genes differentially expressed in CIS CD4^+^ T cells, as determined in the GSE62584 dataset (**B**); network constructed using the 13 genes overlapping the MIF regulatory network and the upregulated genes in CD4^+^ T cell from CIS patients (**C**).

Based on the results from Figure 1, we next wanted to determine whether a significant enrichment of the MIF network could be found in CD4^+^ T cells from patients with CIS. The MIF network comprised 51 genes (Figure 2A). We analyzed the differential transcriptomic feature of the CD4^+^ T cells from patients suffering from CIS and found that 203 and 123 genes were significantly upregulated and downregulated, respectively, as compared to the control donors (Figure 2B). Among the upregulated genes, 13 are also found in the MIF regulatory pathway, i.e., *APRT*, *CLPP*, *CLTA*, *DDT*, *HSPD1*, *MIF*, *MRPL23*, *MZT2A*, *RPL29*, *RPL35*, *RPL36*, *SSSCA1,* and *UQCRQ* (Figure 2C), entailing a strong statistical significance (*p* 0.001). No overlap was observed among the genes belonging to the MIF regulatory pathway and the downregulated genes during the episodes of CIS (Figure 2B).

## 4. Discussion

CIS is a non-infectious inflammatory process observed in young adults that affects the optic nerve, the brainstem, or the spinal cord. Although patients usually recover from the initial episode, several cases of CIS precede conversion to clinically definite MS (CDMS), with the risk of developing MS 15 years after the onset of optic neuritis being 50% [14].

While certain factors including MRI lesions and CSF oligoclonal bands are unequivocally associated to the risk of developing CDMS, other proposed risk factors such vitamin D deficiency, Epstein-Barr virus infection, smoking, HLA genes, and immunological abnormalities are more controversial. The use of MRI to exclude alternative causes and to define the risk for MS has allowed earlier diagnosis and treatment of MS immediately at or soon after CIS presentation [15]. Disease-modifying treatments (DMT) including Interferon beta and glatiramer acetate have been shown to delay the development from CIS to CDMS to some extent, and to potentially be able to dampen further tissue damage, including demyelination and axonal loss. However, the use of DMT in CIS is jeopardized by the doubtful long-term clinical prognosis and risk benefit ratio and occurrence of side effects. Therefore, the main goal of novel therapeutic avenues is to achieve safe and effective long-term DMT [10,11].

In this regard, the better understanding of immunopathogenic pathways operating in CIS are important to design “pathogenetic”-tailored therapeutic approaches that specifically counteract those pathways operating in the development and maintenance of the disease and to identify novel molecules implicated in the pathogenesis of CIS, that may also serve as biomarkers.

In the present study, we evaluated the expression of the cytokines and receptors belonging to the family of cytokine MIF, namely MIF, DDT, and the receptors CD74, CD44, CXCR2, and CXCR4 in patients with CIS by using a publicly available microarray dataset. The use of whole-genome expression data has been extensively used for the identification of novel pathogenic pathways and therapeutic targets in a variety of diseases, e.g., autoimmunity [16,17,18,19], cancer, as well as neurological and psychiatric diseases. The results presented herein demonstrate for the first time that peripheral CD4^+^ T cells from patients with CIS overexpress MIF, DDT, and the receptor CD44 during the course of CIS. Accordingly, a significant overlap was observed between the genes overexpressed in CD4^+^ T cells from patients with CIS and the genes belonging to the MIF regulatory network. This upregulated expression appeared to be unique for CD4^+^ T cells, as other immune cells including CD8^+^ T cells, B cells, and monocytes from these patients exhibited expression levels of these molecules that were superimposable to those observed in healthy controls.

Our data are in agreement with a previous study reporting that serum levels of MIF are higher in progressive patients with CIS than in non-progressive patients [20]. However, increased levels of MIF during CIS were not reported in a recent study [21] reporting that CIS patients experiencing a very rapid onset of CDMS had a 1.5-fold increased CXCR4 and 1.3-fold reduced CD74 surface levels as compared to those with slow or no onset of CDMS, pointing out to a negative correlation between CXCR4 and CD74 levels on B cells in these patients. It is hypothesized the inverse regulation of MIF/CD74 and CXCR4 expression in B cells in CIS patients with rapid development of CDMS may witness the existence of an immature B cell populations with senescent features, that escaped peripheral tolerance [21]. Clearly, the data of Rijvers and coworkers [21] differ from our own present analysis and the previous study indicating increased levels of MIF during CIS [20], as well as with the increasing evidence of upregulated secretion and signaling of MIF and DDT in MS and its rodent counterpart [8,9,22,23]. The reasons for these divergent findings are difficult to explain and remain to be established having in mind the possible occurrence of multiple variables, including sex and age of the patients in the different studies, along with presenting clinical characteristics, including body mass index and eventually misdiagnosed comorbidities. However, since it is known that most cases of MS may depend on the combined action of T cells and B cells [24], the possibility cannot be excluded that deregulation of MIF (and likely DDT) production and signaling may occur simultaneously and in an opposite fashion on CD4^+^ T cells and B cells, with the former being characterized by augmented production of MIF and the latter by a reduced production of MIF. In turn this may activate distinct pathogenetic pathways in T and B cells, leading to development and progression of the disease. This hypothesis is clearly speculative, and it remains to be formally demonstrated. It should however be noted that in rodent models of the disease MIF or DDT deletion, or specific MIF inhibitors, have convergently and independently improved the course of the disease [8,9]. Hence, if the hyporegulated expression of MIF also occurs in rodent B lymphocytes during EAE, their pathogenetic action is not influenced by genetic or pharmacological abrogation of MIF. It is worth mentioning that the CSF levels of MIF have also been studied with divergent results in ON associated with MS. While one study found that CSF levels of MIF in these patients were even higher than those observed in MS relapses [25], the other observed lower levels of MIF in CSF samples of patients with MS-associated ON as compared to MS patients with stable RR-MS [26]. The reason for the discrepancies is not known but may possibly depend on the relatively low number of patients and different ethnicities. It is more difficult to compare the reasons for the discrepancies between our present analysis and the study by Pawlitzki and coworkers [26], as they measured MIF levels in CSF, while we studied peripheral CD4^+^ T cells. Different cells, including glial cells and B cells, may contribute to the intrathecal production of MIF. In addition, the net CSF levels of MIF reflects the ratio between secreted MIF and expressed receptors so that an augmented expression of the receptors intrathecally may consequently reduce even an augmented in situ secretion of MIF. Finally, the conditions studied, though similar from clinical and pathogenetic point of view, are different nosographic entities with our study evaluating patients suffering from ON in the context of CIS as first episode of MS, and the other 2 studies evaluating MS-associated ON in patients with clinically developed MS.

Our observation that MIF DDT and CD44 are hyper-expressed in the peripheral CD4^+^ T cells from patients with CIS concurs with the emerging concept of the major pathogenetic role played from MIF and DDT in MS, and progressive MS [8]. In addition, we have previously demonstrated a pathogenetic role of MIF also in the demyelinating disease of the peripheral nervous system, Guillain Barre’ syndrome (GBS) [27], and its rodent counterpart model, experimental allergic neuritis (EAN) [27], in that MIF levels correlated with disease severity and was prevented by the small molecule inhibitor ISO1 [27]. Taken together, these data seem to indicate that the MIF cytokine family may play a larger than expected role in immunoinflammatory demyelinating processes contributing to MS, GBS, CIS and possibly MS-associated ON.

It is interesting that the overexpression of MIF, DDT and the CD44 receptors occurs in CD4^+^ T (Th) cells from patients with CIS but not in other immune cells including CD8^+^ T cells, B cells and macrophages that exhibit an expression profile of these molecules superimposable to that of healthy controls. It is known that on the basis of their function and different cytokine secretory capacities CD4^+^ T (Th) cells can be subdivided in at least six subclasses that include Th1, Th2, Th3, Th9, Th17 and Treg and the recently described Th1-like Th17 cell subset [28]. We have not presently defined which of these Th subsets overexpress MIF, DDT and the CD44 receptors during CIS. It is also difficult to hypothesize whether the overexpression of MIF family signatures in CD4^+^ T cells of CIS patients reflects the preponderance of a particular CD4 T cell subset during CIS and its eventual pathogenetic contribution. In fact, while several studies have well documented the pathogenetic role of upregulated Th1 and Th17 mediated events in the development and progression of MS [29] much less is known on the role of Th subsets and their cytokines in the pathogenesis of CIS although a reduced levels of the anti-inflammatory cytokines IL-10 and TGF-beta has been reported to be associated with the disease [30,31].

However, identification of precise pathogenetic pathways operating in CIS lies outside the focus of our present phenomenological study. With this caveat in mind, there are several possible modalities by which the upregulated synthesis and function of MIF and DDT may contribute to the pathogenesis of CIS, including the secretion of proinflammatory cytokines and chemokines that could amplify the pathogenetic pathways of CIS provoking recruitment of inflammatory cells through blood brain barrier [6].

Our study represents an additional proof of concept validating the numerous lines of evidences that the MIF cytokine family may possess unique pathogenetic properties, that propose them as key contributors in immunoinflammatory demyelinating diseases. This warrants further studies aimed at the further better identification of MIF family of cytokines in these diseases, along with their possible identification as biomarker tools and therapeutic targets. As stated above, the reduced MIF signature of B cells of RR MS and CIS patients recently reported [21] warrant further studies to be fully integrated into the role of MIF cytokine family in MS and related disorders.

This present data are consistent with previous work by Benedek and coworkers [8] who demonstrated that male patients with progressive MS had augmented blood levels of MIF and DDT levels as compared to both females with progressive MS and MS patients with relapsing-remitting forms of the disease. The increased levels of MIF and DDT levels in males significantly correlated with the presence of two high-expression promoter polymorphisms located in the MIF gene, a -794CATT5-8 microsatellite repeat and a -173 G/C SNP [8]. Knocking out of either MIF or DDT rendered the mice less susceptible to EAE development [8]. In a transcriptomic analysis we observed an upregulation of the receptors involved in MIF signaling both in the animal model and in MS patients along with a significant increase in MIF receptors in the CNS lesions associated to MS. In agreement with the data of Benedek and coworkers [8], we demonstrate that negating the endogenous action of MIF with its specific small molecule inhibitor ISO1 ameliorated the course of the disease as compared to vehicle-treated animals [9].

Although convergent with these multiple lines of evidence on the role of MIF family of cytokines in MS, and possibly related CIS, our analysis bears some intrinsic limitations that primarily include the low number of patients. In addition, accurate clinical and anamnestic information of the patients (BMI, ethnicity) are also lacking as it often occurs in GWAS studies. Nonetheless, our study has also important merits and novelties, including the observation of simultaneous increased peripheral MIF and DDT levels in CIS with rapid conversion to CDMS. In agreement with emerging literature, data on the role of MIF in immunoinflammatory demyelinating diseases these data warrant future studies aimed at deciphering the potential role of MIF cytokine family in CIS and the eventual predictivity of conversion to CDMS in patients with CIS that are high producers of MIF and DDT or bear specific MIF genetic polymorphisms. Along this line, it will be interesting to evaluate in larger groups of patients with CIS whether MIF and DDT levels correlated with the sex of the patients. Proving a pathogenetic role of the MIF cytokine family in CIS would allow us to design novel tailored therapeutic approaches to specifically counteract MIF and DDT in this condition, or at least for some specific forms of them, initially for example for those patients that are poor responders to current early DMT treatment. This could be achieved with some of the several small molecule inhibitors of MIF that are in preclinical development [6]. These include ISO1 which we have shown to be effective as well as EAE and EAN [27], and/or a potent dual small molecule inhibitor of MIF and DDT [32] that could be of particular interest for diseases characterized by combined dysregulated production of MIF and DDT such as CIS. More recently, it has been shown that the biological function of MIF may be inhibited by nitrosylation [33]. This would allow the use of nitric oxide (NO)-hybridized drugs such as NO-aspirin and NO-hybridized antiretroviral protease inhibitor, such as lopinavir, for the treatment of MIF-dependent pathologies including, eventually, MS and CIS. We have indeed shown that Lopinavir-NO exerts stronger immumomodulatory properties in vitro and in vivo than its parental drug [34]. It inhibits the PI3K/Akt/mTOR pathway [35,36] that is implicated in EAE and MS [37], and in an effective fashion prevents the development of a MIF-dependent immunoinflammamtory hepatitis in mice [34,38]. An anti-MIF mAb that has been studied in Phase II PoC in cancer patients as well as anti-CD74 mAb that is in clinical phase and would allow simultaneous blockade of MIF and DDT signaling are of even more relevant for more rapid application in the clinical setting [6].

## 5. Conclusions

Overall, our data suggest that overexpression of the MIF cytokine family signature may occur in CD4^+^ T cells from patients with CIS and that this phenomenon may be implicated in the pathogenesis of the disease, offering the possibility to represent both a diagnostic marker and a therapeutic target.
